# Contrasting response of biomass and grain yield to severe drought in Cappelle Desprez and Plainsman V wheat cultivars

**DOI:** 10.7717/peerj.1708

**Published:** 2016-02-18

**Authors:** Kenny Paul, János Pauk, Zsuzsanna Deák, László Sass, Imre Vass

**Affiliations:** 1 Institute of Plant Biology, Biological Research Centre, Szeged, Hungary; 2 Deparment of Biotechnology, Cereal Research Nonprofit Company, Szeged, Hungary

**Keywords:** Drought stress, *Triticum aestivum* L, Variable chlorophyll fluorescence, CO_2_ gas exchange measurements, P700 absorbance, Cyclic electron flow (CEF), Biomass, Grain yield

## Abstract

We report a case study of natural variations and correlations of some photosynthetic parameters, green biomass and grain yield in Cappelle Desprez and Plainsman V winter wheat (*Triticum aestivum* L.) cultivars, which are classified as being drought sensitive and tolerant, respectively. We monitored biomass accumulation from secondary leaves in the vegetative phase and grain yield from flag leaves in the grain filling period. Interestingly, we observed higher biomass production, but lower grain yield stability in the sensitive Cappelle cultivar, as compared to the tolerant Plainsman cv. Higher biomass production in the sensitive variety was correlated with enhanced water-use efficiency. Increased cyclic electron flow around PSI was also observed in the Cappelle cv. under drought stress as shown by light intensity dependence of the ratio of maximal quantum yields of Photosystem I and Photosystem II, as well by the plot of the Photosystem I electron transport rate as a function of Photosystem II electron transport rate. Higher CO_2_ uptake rate in flag leaves of the drought-stressed Plainsman cv. during grain filling period correlates well with its higher grain yield and prolonged transpiration rate through spikes. The increase in drought factor (DFI) and performance (PI) indices calculated from variable chlorophyll fluorescence parameters of secondary leaves also showed correlation with higher biomass in the Cappelle cultivar during the biomass accumulation period. However, during the grain filling period, DFI and PI parameters of the flag leaves were higher in the tolerant Plainsman V cultivar and showed correlation with grain yield stability. Our results suggest that overall biomass and grain yield may respond differentially to drought stress in different wheat cultivars and therefore phenotyping for green biomass cannot be used as a general approach to predict grain yield. We also conclude that photosynthetic efficiency of flag and secondary leaves is correlated with grain yield and green biomass, respectively. In addition, secondary trait associated mechanisms like delayed senescence and higher water-use efficiency also contribute to biomass stability. Our studies further prove that photosynthetic parameters could be used to characterize environmental stress responses.

## Introduction

Drought is a complex environmental stress factor, which significantly affects the growth and development of plants. The effects of drought are expected to increase with climate change and increasing water shortage. Plants have evolved specific acclimation and adaptation mechanisms in order to cope with short- and long-term limitation of water availability. These mechanisms depend on species, genotypes and the co-occurrence with other stresses, such as high temperature or evaporative demand. Analysis of these protective mechanisms can be a key for improved understanding of the molecular background of drought stress tolerance and resistance (Harb et al., 2010; Berger, Parent & Tester, 2010).

Plants can respond to limited soil water availability by various strategies including drought escape, which is described as the ability of plants to complete their life cycle before severe stress sets in. Besides the escape strategy plants can resist water scarcity conditions via drought avoidance, or drought tolerance (Levitt, 1980; Price et al., 2002). Drought avoidance is classified as the ability of plants to maintain high tissue water potential despite soil water deficit. This can be achieved via improved water uptake under stress. The capacity of plant cells to hold acquired water and reduce water loss also confers drought avoidance. Plants can survive water stress by improved root traits, decreasing stomatal conductance, leaf area and radiation absorptivity (Price et al., 2002). Drought tolerance on the other hand is the ability of plants to utilize limited amount of water, leading to low tissue water potential, with higher efficiency regarding growth, biomass accumulation and reproduction (Ingram & Bartels, 1996). Plants under drought stress accumulate compatible solutes and thrive on by maintaining cell turgor and reducing evaporative water loss (Yancey et al., 1982).

In different species it has been shown that drought conditions affect the relationship between the carbon content in photosynthetic organs, such as leaves (source), and the carbon content in heterotrophic organs, such as seeds and roots (sink), indicating that the processes related to carbon partitioning are sensitive targets of drought stress (Cuellar-Ortiz et al., 2008). These alterations cause the abortion of reproductive structures, as well as a decrease in the accumulation of biomass in storage organs, causing losses in crop production (Boyer & Westgate, 2004; Marcelis et al., 2004).

For optimizing cereal crop productivity under drought stress, it is highly important to characterize and understand the relationship between the responses induced by water limitation at the level of green biomass accumulation and seed production. Precision phenotyping is a rapidly growing field of plant sciences, which provides excellent tools for quantitative characterization of the adverse consequences of various stress effects including drought (Berger, Parent & Tester, 2010; Golzarian et al., 2011). Phenotyping approaches vary from manual platforms to complex robotic systems with automated data acquisition and measurement workflows. They usually comprise non-invasive measurements at a spatial resolution stretching from the sub cellular level to canopy stands, and temporal resolutions ranging from seconds to entire growing seasons (Dhondt, Wuyts & Inzé, 2013). Despite the obviously very high potential of plant phenotyping to characterize the consequences of stress-induced effects, there is a highly important and often overlooked question in the case of cereal crops: whether the most easily quantified phenotypic parameters such as the above ground green biomass can predict correctly the grain yield or not.

In the present work we aimed at the characterization of drought-induced responses of two model wheat cultivars, Plainsman V and Cappelle Desprez, both of them are winter wheat varieties (http://genbank.vurv.cz/wheat/pedigree). Plainsman V, released in 1974 in Kansas, USA, produces a moderate yield of high-quality grain. Cappelle Desprez, which gives a high yield under intensive growth conditions, was bred and released in France in 1946. These varieties, are characterized as either being drought tolerant (Plainsman), or drought sensitive (Cappelle Desprez), respectively (Guóth et al., 2009; Secenji et al., 2010).

Our data obtained by measurements of PSI and PSII electron transport, thermal imaging of leaf temperature, as well as phenotypic characterization of biomass accumulation suggest that under drought stress biomass development is correlated with photosynthetic efficiency of secondary leaves, as well as secondary trait associated mechanisms like delayed senescence and lower transpiration rate. However, grain yield stability under drought stress is higher for the Plainsman cultivar, which has smaller biomass but larger grain production, than the Cappelle Desprez cultivar, which can accumulate larger green biomass, but has smaller grain yield. Therefore, in cases when optimization of grain yield is an important target of phenotyping the consequences of drought and other stress effects should include not only the green biomass, but also grain production.

## Methods

### Plant material and growth conditions

Vernalization of one-week old seedlings was carried out for 6-week, at 4 °C in a cold chamber, under continuous dim light. Vernalized plantlets of the Cappelle Desprez and Plainsman V (Guóth et al., 2009) (http://genbank.vurv.cz/wheat/pedigree) winter wheat (*Triticum aestivum* L.) varieties were planted in a soil-sand-peat mixture (3:1:1, v/v/v). Plants were regularly irrigated and grown in controlled green-house conditions for two weeks before starting the drought stress treatment. Photosynthetically active radiation (PAR) within controlled environment was maintained with a 14 h photoperiod at a PPFD of 400–500 µmol m^−2^ s^−1^, 22–25 °C and ca. 45–55% relative humidity.

### Drought stress treatment and measuring protocol

Drought stress was induced on the above mentioned seedlings (4–5 leaf stage) by limiting irrigation to ensure 10% field capacity of the soil using the computer-controlled water supply system of our phenotyping platform (Cseri et al., 2013) for a period of 35 days. The control well watered plants were irrigated to keep 60% field capacity of the soil. Biomass accumulation in the vegetative phase, i.e., in the first 3 weeks of the drought treatment, was monitored from the younger fully developed leaves, which are denoted as ‘*secondary leaves.’* While in the reproductive grain filling phase, in the 4th–5th week of the drought treatment, the measurements were performed on the last fully developed leaf, denoted as *‘flag leaf.’* Six replicates of each treatment were used for the study in three separate experimental trials conducted in August–September 2012, April–May and July–August 2013, respectively.

### Gas exchange measurements

Gas exchange parameters: CO_2_ uptake rate, transpiration, stomatal conductance and intercellular CO_2_ concentration were measured by using a Licor 6400 gas analyzer (LI-COR Biosciences, Lincoln, NE, USA). Two to three selected pieces of attached secondary, as well as flag leafs from plant replicates treated under respective drought regimes were inserted into the gas cuvette for individual measurements. The gas cuvette conditions were set to 400 ppm CO_2_, ambient temperature and growth light intensity of photosynthetically active radiation (of 400 µmol m^−2^ s^−1^).

Water-use efficiency (WUE) was calculated from the ratio of photosynthesis (A) and transpiration (E) (Farquhar & Richards, 1984; Lovelli et al., 2007).

### OJIP chlorophyll *a* fluorescence measurements

OJIP chlorophyll *a* fluorescence transients were measured by a Plant Efficiency Analyzer (Pocket Pea, Hansatech, UK). The transients were induced by red light from an LED source (627 nm, up to 3,500 µmol m^−2^ s^−1^ intensity). Prior to measurements, performed on the adaxial surface, leaves were dark adapted to for 20 min using light tight leaf-clips. The OJIP-test (Strasser, Srivastava & Tsimilli-Michael, 2000) was used to analyze the chlorophyll *a* fluorescence transients and the following original data were acquired: O (*F*_*o*_) initial fluorescence level (measured at 50 µs), P (*F*_*m*_) maximal fluorescence intensity, as well as the J (at about 2 ms) and the I (at about 30 ms) intermediate fluorescence levels. From these specific fluorescence features the following parameters of photosynthetic efficiency were calculated: maximal PSII quantum yield, *F*_*v*_/*F*_*m*_; The ratio of variable fluorescence to initial fluorescence, *F*_*v*_/*F*_*o*_ where *F*_*v*_ = *F*_*m*_ − *F*_*o*_; Probability of electron transport out of *Q*_*A*_, (1-*V*_*j*_)/*V*_*j*_ where *V*_*j*_ = (*F*_2ms_ − *F*_*o*_)/*F*_*v*_; Total complementary area between the fluorescence induction curve and *F*_*m*_ of the OJIP curve, Area; *Q*_*A*_ reducing reaction centers per PSII antenna chlorophyll, RC/ABS = (*F*_*V*_∕*F*_*M*_) ⋅ (*F*_*J*_ − *F*_0_)/[4 ⋅ (*F*_300μs_ − *F*_0_)] (Zurek et al., 2014; Campos et al., 2014; Strasser, Tsimilli-Michael & Srivastava, 2004); Performance index (potential) for energy conservation from photons absorbed by PSII to the reduction of intersystem electron acceptors, PI_Abs_ (Zivcak et al., 2008; Campos et al., 2014).

\begin{eqnarray*}{\mathrm{PI}}_{\mathrm{Abs}}= \frac{1-({F}_{o}/{F}_{m})}{{M}_{o}/{V}_{j}} \times \frac{{F}_{m}-{F}_{o}}{{F}_{o}} \times \frac{1-{V}_{j}}{{V}_{j}} \end{eqnarray*}

where *M*_*o*_ = 4∗ (*F*_300μs_ −*F*_*o*_)/ (*F*_*M*_ − *F*_*o*_) represents initial slope of fluorescence kinetics.

For screening of drought stress tolerance a further parameter, the so-called drought factor index (DFI) was used, which is derived from PI values measured after 1 or 2 weeks of drought treatment, and reflects the ability of plants to tolerate sustained drought stress conditions (Oukarroum et al., 2007)

\begin{eqnarray*}(\mathrm{DFI})=\log ({\mathrm{PI}}_{\mathrm{week}1}/{\mathrm{PI}}_{\mathrm{control}})+2\log ({\mathrm{PI}}_{\mathrm{week}2}/{\mathrm{PI}}_{\mathrm{control}}). \end{eqnarray*}

### Simultaneous measurements of P700 redox state and chlorophyll fluorescence

Variable chlorophyll fluorescence from PSII and the amount of oxidized PSI primary Chl electron donor (P700^+^) was simultaneously measured using a DUAL-PAM-100 system (WALZ, Effeltrich, Germany). From the fluorescence data *F*_*v*_/*F*_*m*_ and the effective quantum yield of photochemical energy conversion in PS II, Y(II)= (*F*_*m*′_ − *F*)/ *F*_*m*′_ (Genty, Briantais & Baker, 1989) where *F*_*o*_, *F*_*o*′_ are dark fluorescence yield from dark- and light-adapted leaf, respectively and *F*_*m*_, *F*_*m*′_ are maximal fluorescence yield from dark- and light-adapted leaf, respectively, were calculated. The P700^+^ signal (P) may vary between a minimal (P700 fully reduced) and a maximal level (P700 fully oxidized). The maximum level of P700^+^ is called *P*_*m*_ in analogy with *F*_*m*_. It was determined by application of a saturation pulse (300 ms, 10,000 µmol m^−2^ s^−1^; 635 nm) after pre-illumination with far-red light. *P*_*m*′_ is analogous to the fluorescence parameter *F*_*m*′_ and was determined by applying 800 ms saturating pulse of 635 nm red light. The photochemical quantum yield of PSI, Y(I) is the quantum yield of photochemical energy conversion in PSI. It is calculated as Y(I) = (*P*_*m*′_ − *P*)/ *P*_*m*_. Y(ND) is the quantum yield of non-photochemical energy dissipation in PSI due to donor side limitation, Y(ND) =*P*∕*P*_*m*_. Y(NA) is the quantum yield of non-photochemical energy dissipation due to acceptor side limitation in PSI, Y(NA) = (*P*_*m*_ − *P*_*m*′_)/*P*_*m*_, and Y(I) + Y(ND) + Y(NA) = 1 (Klughammer & Schreiber, 1994; Singh et al., 2014). Non-photochemical quenching NPQ (Bilger & Bjorkman, 1990), was calculated as (*F*_*m*_ − *F*_*ms*_)/*F*_*ms*_, where *F*_*m*_ represents the fluorescence of a dark-adapted sample and *F*_*ms*_ represents a fluorescence of the illuminated sample. Plants were dark-adapted for ∼20 min and kinetics were measured after repeated light pulses of 94 PPFD for 300 s. Leaves were subsequently relaxed in darkness for 240 s and fluorescence while continuously measuring and recording fluorescence (Szalonek et al., 2015).

The electron transport rates through PSII as well as through PSI were determined simultaneously (Miyake et al., 2005; Fan, 2007). The apparent rate of electron transport in higher plants were calculated as ETR(II) = Y(II) ∗ PPFD ∗ 0.5 ∗ 0.84 and ETR(I) = Y(I) ∗ PPFD ∗ 0.5 ∗ 0.84 (Genty, Briantais & Baker, 1989), where Y(II) and Y(I) are effective quantum yields of PSII and PSI respectively, PPFD is the photon flux density of incident photosynthetically active radiation and two coefficients (0.5 and 0.84 for higher plants, which imply that PSII and PSI are equally excited, and that due to leaf absorbance properties only 84% of incident irradiance will be absorbed by the photosystems, respectively (Björkman & Demmig, 1987; Schreiber, 2004).

### Thermal imaging

Thermal images were taken by using a Thermo Varioscan (Jenoptik, Laser optik, Systeme, GmbH) camera as described by Kana & Vass (2008). Thermal images of wheat cultivars under various drought stress treatments were analyzed by using ImageJ software to select and measure areas based on color. Images were threshold using Hue, Saturation and Brightness (HSB) color space and converted to binary values by defining a color scale cutoff point. Values of evaporative cooled area, represented by pixels below the ambient temperature, become black and those in above become white.

### Digital imaging

Digital images of seedlings were taken by using a Nikon D80 camera equipped with an AF-S DX Zoom-NIKKOR 18–135 mm objective (f/3.5–5.6G ED-IF Lens) and close-up rings. Digital images of plant replicates under various drought stress treatments were analyzed for green biomass area using ImageJ software. We used colour thresholding to select just the plant green area and exclude the stand, pot, shadows and yellowish leaves (http://rsbweb.nih.gov/ij/).

### Statistical analysis

The comparison of traits of plants of the same variety, which were grown under different watering conditions was based on the two-sample Student’s *t*-Tests (http://www.physics.csbsju.edu/stats/t-test.html). Levels of significance (*P* values) in differences from means of well watered and drought stressed plants are indicated in the figure legends.

## Results

### Phenotyping for biomass accumulation and grain yield

**Figure 1:**
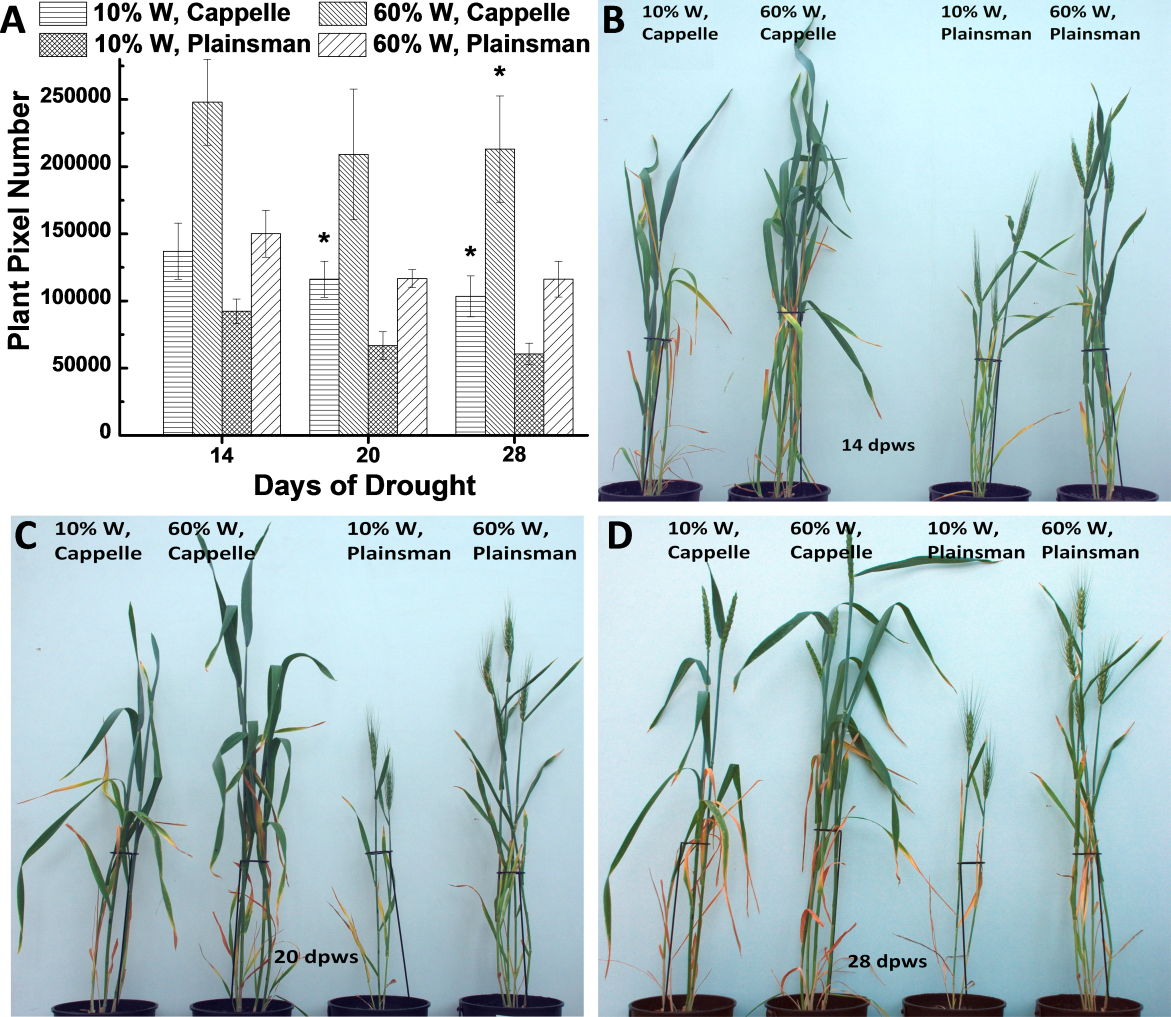
The effect of drought stress on the accumulation of green biomass. (A) The area of the green leaves and shoots, which is used as a proxy for the above ground green biomass, was calculated from the analysis of digital photographs for the Cappelle Desprez and the Plainsman V wheat cultivars kept either under well watered (60% field capacity) or water limited (10% field capacity) conditions. The measurements were performed after 14, 20 and 28 days following the start of the drought treatment, which occurred after two weeks of planting the vernalized seedlings into pots. The means ± SE were calculated from five plants/treatment. The asterisks indicate significant differences (^∗^*p* ≤ 0.05) between plants of the two different varieties, which were kept at the same soil water content. (B–D) show representative pictures of Cappelle Desprez and Plainsman V wheat plants after 14, 20 and 28 days of water stress, respectively.

Growth of wheat plants was monitored by digital photography by recording green pixel-based shoot surface area of wheat plants, which was performed during the whole growth period once a week. According to our previous data the green pixel-based shoot surface area correlates with green biomass (Fehér-Juhász et al., 2014). Digital RGB imaging of leaf/shoot area showed that the Cappelle Desprez cv. produces larger above ground green biomass than Plainsman V not only under conditions of water availability but also under water scarcity (Fig. 1). This conclusion is supported by direct measurement of green biomass (Fig. S1).

The grain yield data showed an opposite trend compared to the biomass accumulation, i.e., although grain yield under well watered conditions was higher in the Cappelle Desprez cv., the Plainsman V produced more grains under water limitation, and showed lower grain yield loss (24%) than the Cappelle Desprez (74%) (Table 1), see also Fig. S2.

**Table 1:** Effect of drought stress on the total yield of the experiment and the thousand-kernel weight of Cappelle Desprez and Plainsman V wheat cultivars.

Treatment	Total grain yield/plant^a^ (g)	1,000 kernel weight^a^ (g)
10% W, Cappelle Desprez	0.41 ± 0.01 (^∗∗^*p* < 0.01)	19.18 ± 0.32 (^∗∗∗^*p* < 0.001)
60% W, Cappelle Desprez	1.57 ± 0.12 (^∗^*p* < 0.05)	41.20 ± 1.47 (^∗∗∗^*p* < 0.001)
10% W, Plainsman V	0.77 ± 0.06	29.10 ± 1.79
60% W, Plainsman V	1.01 ± 0.07	32.14 ± 0.91

**Notes.**

a Data is average of three replications.

The ∗ signs indicate the level of significance for the difference between the two cultivars when compared under the same watering conditions.

### Carbon fixation, stomatal functions and water-use efficiency

**Figure 2:**
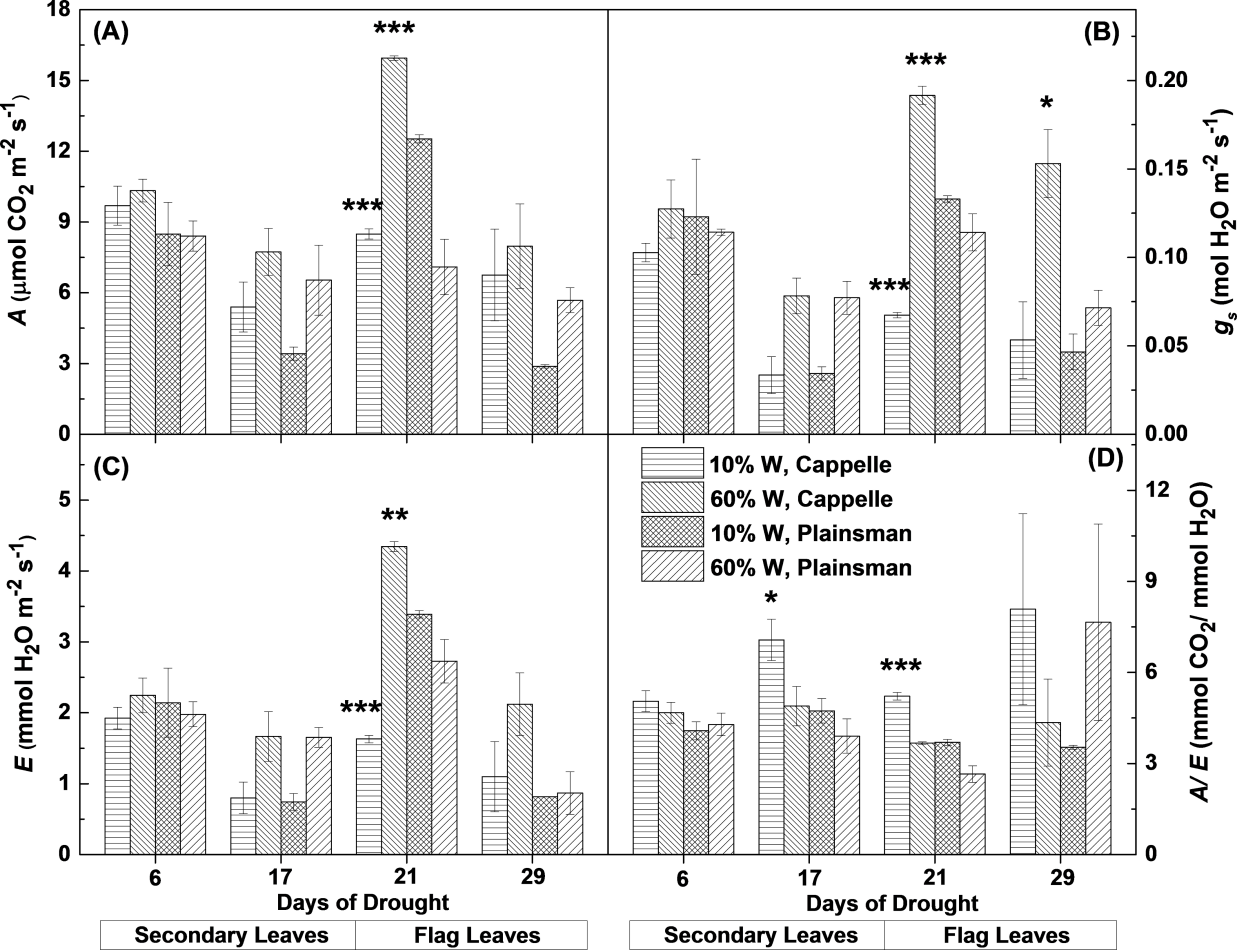
Gas exchange parameters of Cappelle Desprez and Plainsman V wheat plants. (A) Net rate of photosynthesis (*A*). (B), Stomatal conductance (*g*_*s*_). (C), Transpiration rate (*E*). (D) Water-use efficiency (*A*∕*E*). Measurements were performed in well watered (60% W) or water limited (10% W) wheat plants. Values represent means ± SE of 5 plants/treatment. The asterisks indicate significant differences (^∗^*p* ≤ 0.05, ^∗∗^*p* ≤ 0.01, ^∗∗∗^*p* ≤ 0.001) between plants of the two different varieties, which were kept at the same soil water content.

From gas exchange measurements, we could observe that the net CO_2_ uptake rate and other gas exchange parameters were not affected in the first week of drought stress. After the second week the net CO_2_ uptake decreased in the secondary leaves of water limited plants both in the case of drought sensitive Cappelle Desprez and drought tolerant Plainsman V cv. (Fig. 2A). Interestingly, in the grain filling period only Cappelle Desprez cv. responded with decreased CO_2_ uptake to the decreased soil water content in case of the flag leaves (Fig. 2A).

The response of stomatal conductance (Fig. 2B) and evaporation rate (Fig. 2C) shows similar pattern to that of CO_2_ uptake. Interestingly, in case of secondary leaves both parameters decreased under drought stress in both cultivars, while in the flag leaves only the Cappelle Desprez cv. showed significant decrease of stomatal conductance and evaporation rate under drought conditions relative to their well watered controls. The Plainsman cv. did not close its stomata in the flag leaves and did not decrease its evaporation rate during early grain filling phase (21st day). However, in the later phases (29th day) of the drought stress the stomatal conductance and evaporation showed a decreasing tendency also in the Plainsman V cv. (Figs. 2B and 2C).

The ability of leaves to achieve optimal photosynthesis relative to the amount of used water, i.e., to conserve water under drought conditions, is characterized by the water-use efficiency (WUE), which is given by the ratio of the rates of net photosynthesis and transpiration. In the Cappelle Desprez cv. the WUE (A/E) parameter increased both in the secondary and flag leaves, while in the Plainsman cv. WUE increased only slightly in the secondary leaves and also in the flag leaves during the grain filling phase in the drought stressed plants (Fig. 2D), which is consistent with sustained photosynthesis and flag leaf transpiration in order to maintain high grain yield.

**Figure 3:**
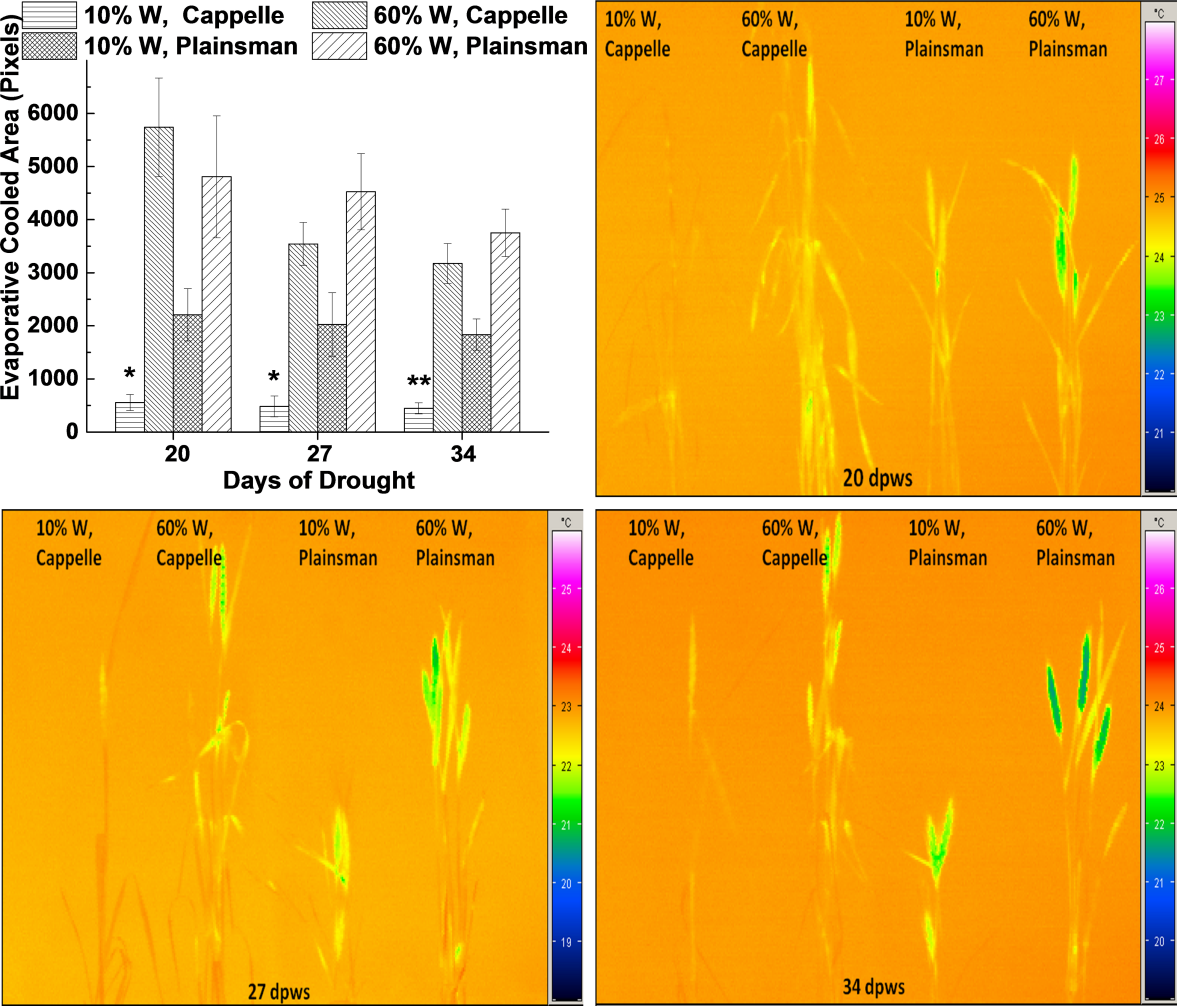
Thermal imaging of drought stressed Cappelle Desprez and Plainsman V wheat plants. (A) Thermal images of Cappelle Desprez and Plainsman V wheat plants were quantified by using ImageJ software by thresholding evaporative cooled area relative to the temperature of the surrounding air. The presented data were obtained from the average of thermal images taken under conditions of water stress (10% W) and well watered control (60% W) on the 20th, 27th and 34th day of drought stress. The means ± SE were calculated from 5 plants/treatment. The asterisks indicate significant differences (^∗^*p* ≤ 0.05, ^∗∗^*p* ≤ 0.01) between plants of the two different varieties, which were kept at the same soil water content. (B–D) show representative thermal images of Cappelle Desprez cv. and Plainsman V cv. plants under water stress (10% W) and control (60% W) conditions taken after 20, 27 and 34 days of drought stress, respectively.

### Thermal imaging

Leaf temperature can be conveniently monitored by thermal imaging in the infrared spectral range, and drought stress induced leaf temperature changes can be studied. The status of stomata influences not only CO_2_ uptake, but also the efficiency of evaporating water from the leaf tissue, which in turn affects the temperature of the leaves. Thermal images were quantified based on pixel numbers calculated from thresholded evaporative cooled area relative to ambient background temperature (Fig. 3A). The data show that the leaf and shoot area, which is cooler than the surrounding air, i.e., cooled by evaporation via transpiration, is small in drought stressed Cappelle Desprez plants indicating low transpiration rate due to stomatal closure (Fig. 3A). In contrast, the Plainsman V cv. has larger cooled leaf area not only in the well watered, but also in the drought stressed plants (Fig. 3A). Evaporative cooling is especially pronounced in the spikes of Plainsman V plants (Figs. 3B–3D), which is in agreement with the open status of the stomata allowing efficient CO_2_ uptake and large net photosynthesis rate for a sustained period during grain filling leading to increased grain yield.

**Figure 4:**
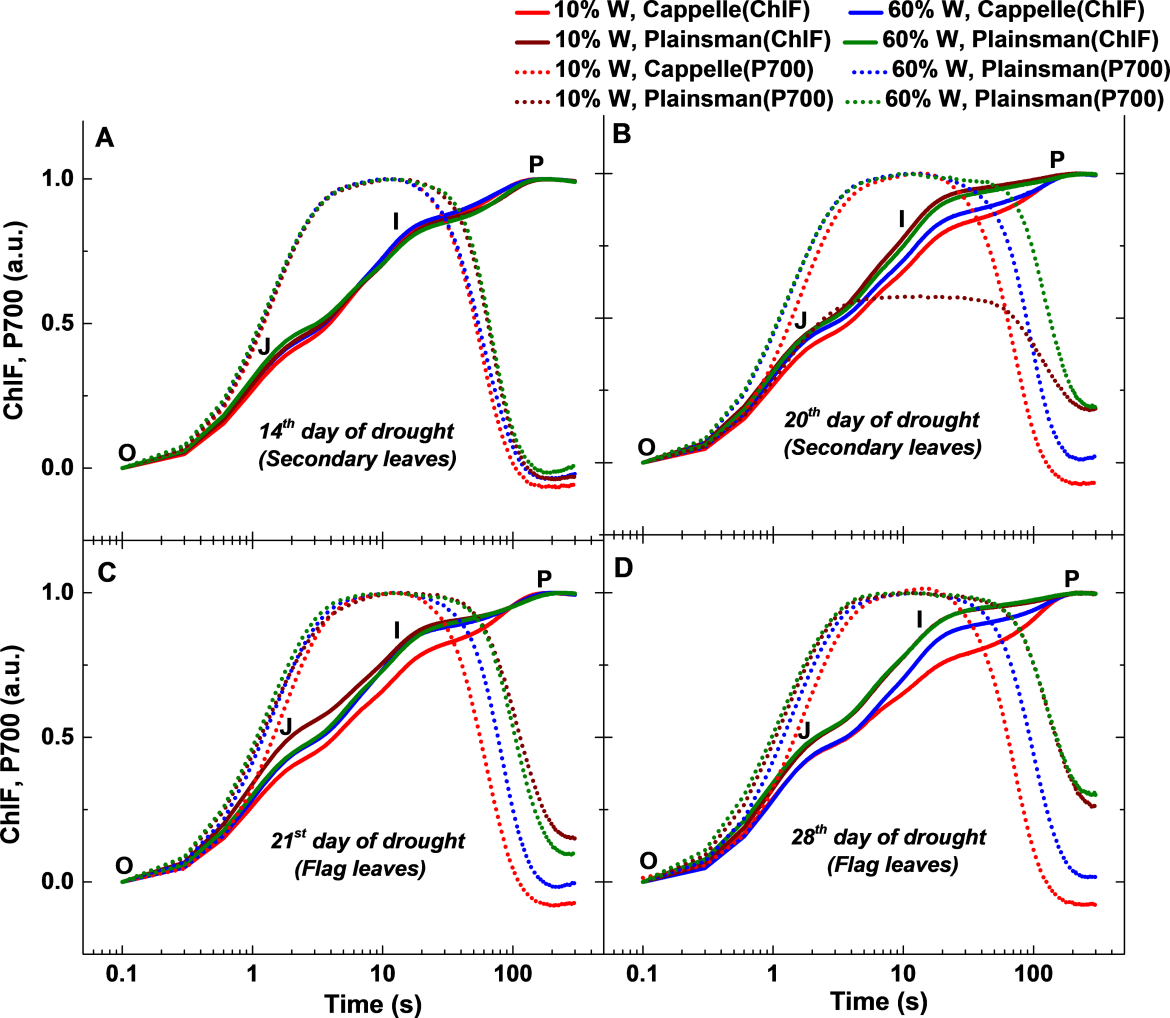
Fast chlorophyll *a* fluorescence and P700 kinetics. The measurements were done in leaves of well-watered (60% W) and drought stressed (10% W) of Cappelle Desprez and Plainsman V cv. wheat plants. Measurements were carried out in early developed secondary leaves on the 14th and 20th days of treatment, while on the 21st and 28th days of treatment in flag leaves. The saturation pulse intensity was 10,000 µmol photons m^−2^ s^−1^ for 0.8 s. The O, J, I and P points represent the standard phases of chlorophyll *a* fluorescence transients.

### Simultaneously recorded fast Chl fluorescence and P700 redox kinetics

We observed drought induced changes in photosynthetic electron transport rate from the analysis of fast chlorophyll and P700 kinetics measured in dark adapted leaves (Fig. 4). The effects of drought on the initial processes of photosynthesis which take place in the Photosystem II complex were characterized by measuring variable chlorophyll fluorescence induction transients, the so called OJIP curves. The measurements were performed both on the secondary leaves, which are expected to reflect photosynthetic activity that is responsible for overall green biomass growth, as well as on flag leaves, which primarily support grain development. The variable fluorescence transients show clear differences in the I-P amplitude, reflects the size of the electron acceptor pool of PSI (Schansker, Tóth & Strasser, 2005; Tsimilli-Michael & Strasser, 2008), and was correlated with a higher PSI activity due to an increased PSI/PSII ratio (Ceppi et al., 2012). In case of secondary leaves, the Cappelle Desprez cv. responded to water withdrawal with only minor changes of the OJIP fluorescence and P700 kinetic transients in the first 2 weeks of the drought treatment (Fig. 4A). After 3 weeks, however, faster rise in Chl fluorescence and P700 oxidation and re-reduction transient in normalized curve was observed in drought stressed Cappelle cv. showing reduction of ferredoxin and increased PSI content (ca. 20%, Fig. 4B) (Schansker, Tóth & Strasser, 2005; Ceppi et al., 2012). In drought stressed Plainsman V we could observe a faster rise of the J-I phase, PSI oxidation was prolonged and PSI re-reduction did not reach normal level. Faster decay of drought stressed Plainsman V shows less functional PSII activity and smaller PSI content. Well-watered controls of both cultivars showed comparable responses in Chl fluorescence and P700 redox kinetics (Fig. 4B).

During the early phase of spiking (21st and 28th day of drought), 10% W Cappelle Desprez cv. showed similar trend of higher PSI content in the flag leaves with an increase of I-P amplitude in fast chlorophyll fluorescence and of P700^+^/P700 ratio kinetics (Figs. 4C and 4D). In drought-stressed Plainsman V we could observe a slower rise of J-I phase and I-P amplitude with increased P700 oxidation ending in faster re-reduction decay kinetics (Figs. 4C and 4D). This indicates higher functional PSII activity for drought stressed Plainsman V in the flag leaves during grain filling period.

### Chl fluorescence parameters reflect plant responses to drought stress

Various biophysical parameters derived from Chl a fluorescence transient measurements help to understand the energy flow through PSII and provide useful indicators of the development and severity of stress effects (Kenny et al., 2011), including drought. One of the useful calculated parameters is the so called performance index (PI), which combines the three main functional steps taking place in PSII (light energy absorption, excitation energy trapping, and conversion of excitation energy to electron transport), and was used as measure of drought stress tolerance (Strauss et al., 2006). PI, which provides useful and quantitative information about the physiological conditions and the vitality of plants, revealed differences among varieties under conditions of drought stress (Oukarroum et al., 2007). Other calculated parameters like Area and RC/ABS also responded for secondary and flag leaves under severe drought stress (Campos et al., 2014).

**Figure 5:**
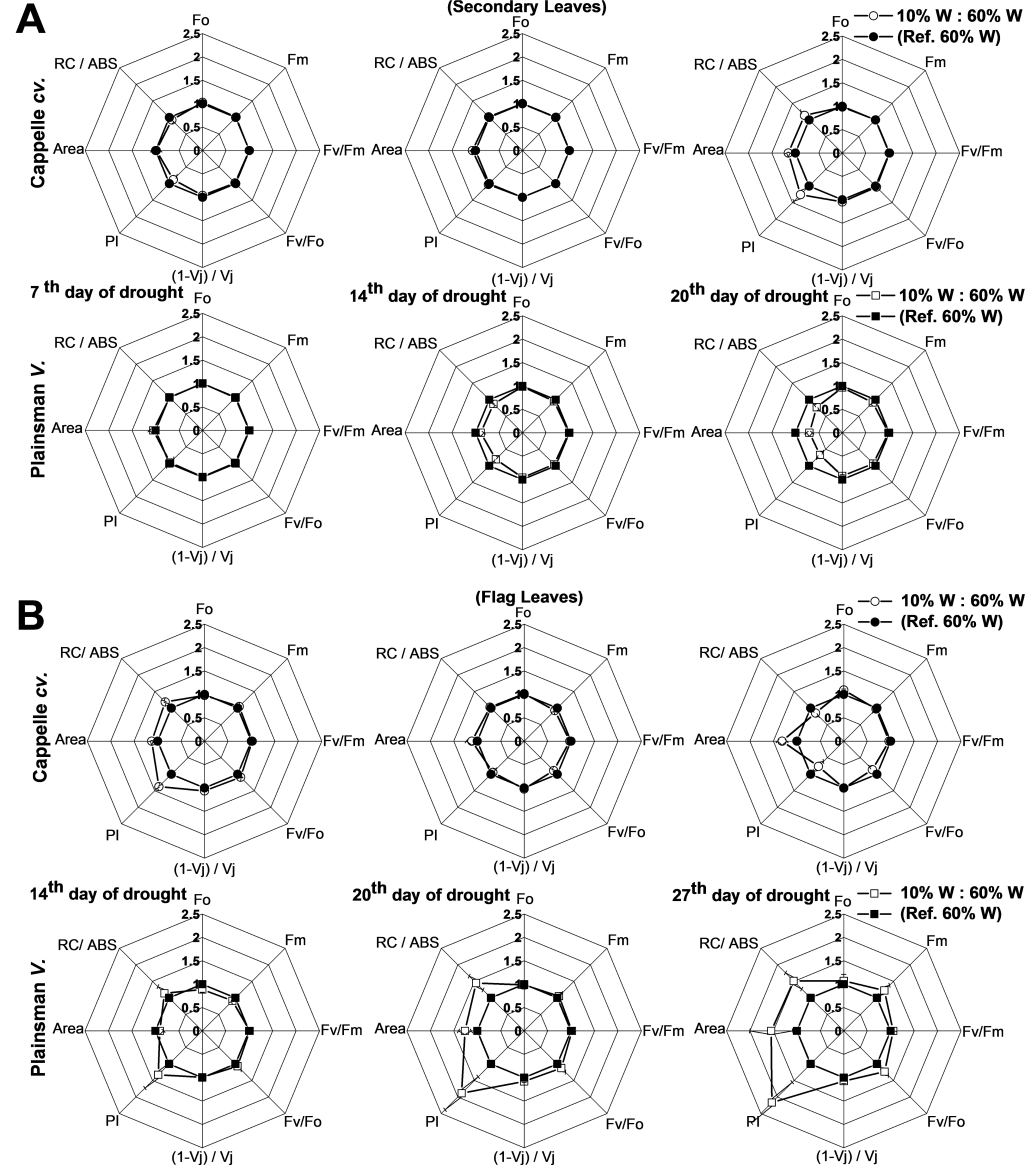
Deduced variable Chl *a* fluorescence parameters. (A) Spider graphs of various chlorophyll fluorescence parameters (*F*_*o*_, *F*_*m*_, *F*_*v*_/*F*_*m*_, *F*_*v*_/*F*_*o*_, (1 − *Vj*)/*Vj*, PI, Area, RC/Abs) are shown in the early developed secondary leaves (A) and in flag leaves (B). Values obtained for drought stressed plants are shown after normalization to their respective well watered controls. Data represent means ± SE of five plants/ treatment.

In case of Cappelle Desprez cv., the secondary leaves responded to water withdrawal with only minor changes of the deduced electron transport parameters in the first 2 weeks of the drought treatment (Fig. 5A). In contrast, the Plainsman V showed a clear tendency for decreasing the PI, the Area reflecting the size of oxidized PQ pool, and the RC/ABS parameters indicating a decreased photosynthetic performance at the level of secondary leaves (Fig. 5A). As regards deduced fluorescence parameters, there was no significant effect of drought stress in either of the developmental stages of the secondary leaves of the Cappelle Desprez cv. with the exception of a small increase of PI after 2 weeks of drought stress (Fig. 5B). In contrast, the Plainsman V cv. showed a marked increase of the PI parameter with the progress of drought stress with some increase also in the Area, RC/ABS, and *F*_*v*_∕*F*_*m*_ parameters after 4 weeks of drought stress (Fig. 5B). These data show that the photosynthetic efficiency of flag leaves of the Plainsman V cv. is affected by drought stress conditions to a smaller extent than of the Cappelle Desprez variety.

For the characterization of drought tolerance on the basis of Chl fluorescence data Strasser and coworkers have introduced the so called drought factor index (DFI). This parameter represents the relative decrease of the performance index (PI) during water scarcity and reflects the ability of plants to tolerate long-term drought stress (Oukarroum et al., 2007). A large positive value of DFI indicates drought tolerance, while a large negative value is characteristic for drought sensitivity. According to Table 2, in the periods of severe drought and senescence the secondary leaves of the Cappelle Desprez and Plainsman V cv. show positive and negative DFI, respectively, which is in agreement with the larger green leaf area and biomass of Cappelle Desprez as compared to that of Plainsman V Interestingly, the DFI values of the flag leaves show an opposite trend with positive DFI for the Plainsman and negative DFI for the Cappelle Desprez cv. Again, this behavior is consistent with the higher grain yield of Plainsman V as compared to the Cappelle Desprez cv. under drought stress.

**Table 2:** Calculated Drought Factor Index (DFI) values of drought sensitive Cappelle Desprez cv. and drought tolerant Plainsman V under medium to severe drought.

	Secondary leaves (DFI)			Flag leaves (DFI)		
Wheat cv./ drought stress	Medium 7–14 *(dpws)*	Severe 14–20 *(dpws)*	Senescence 20–27 *(dpws)*	Medium 14–20 *(dpws)*	Severe 20–27 *(dpws)*	Senescence 27–35 *(dpws)*
Cappelle Desprez	−0.0659	0.1629	0.1870	0.0994	−0.2096	−0.1364
Plainsman V	−0.2548	−0.3489	−0.4978	0.3952	0.7427	0.3099

**Notes.**

The DFI values were calculated from the variable Chl fluorescence transients as described in the Materials and methods in Cappelle Desprez and Plainsman V wheat plants, which were exposed to drought from 7 to 35 days of post water stress (*dpws*).

### Drought induced cyclic electron flow around PSI

A characteristic response of drought stressed wheat plants is the induction of cyclic electron flow (CEF) around Photosystem I (Johnson, 2011; Zivcak et al., 2013; Zivcak et al., 2014), which directs electrons from the acceptor side of PSI back to the PQ pool. This effect is considered as a defense mechanism against oxidative stress that develops under conditions of limited availability of CO_2_ as final electron acceptor due to stomatal closure (Golding & Johnson, 2003; Zulfugarov, Mishra & Lee, 2010). Evidence for cyclic flow at high light exists in all the plants to a greater or lesser extent (Kono, Noguchi & Terashima, 2014).

**Figure 6:**
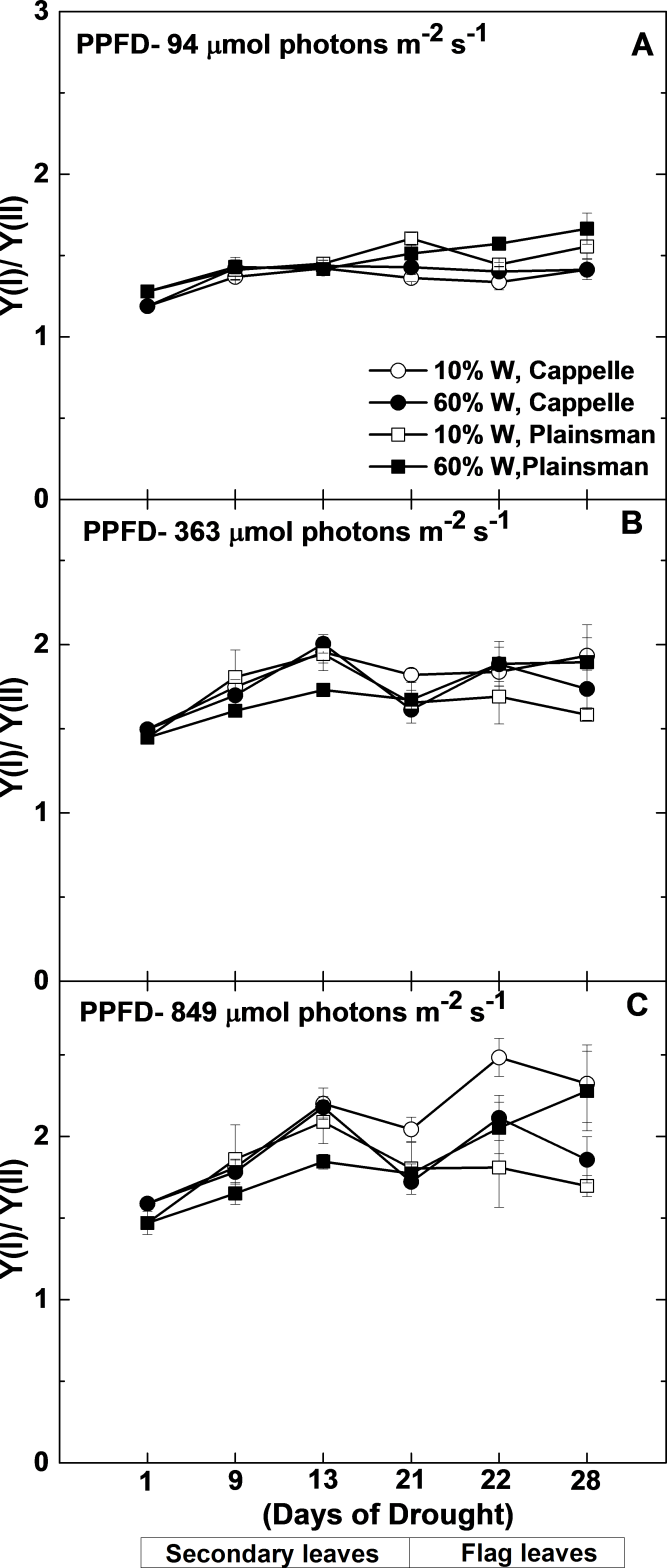
Changes in the Y(I)/Y(II) ratio. Maximal quantum yield of PSI (Y(I)) and PSII (Y(II)) were measured in leaves of well watered (60% W—filled symbols) and severe drought-stressed (10% W—open sysmbols) Cappelle Desprez (circles) and Plainsman V (squares) cv. wheat seedlings at different light intensities of 94, 363 and 849 µmol photons m^−2^ s^−1^. The values, which are shown as a function of the duration of drought treatment, represent the mean ± SE of five plants per treatment.

Since under conditions of cyclic electron flow part of the electrons, which arise from PSII circulate around PSI the rate of electron flow though PSII (ETR(II)) and PSI (ETR(I)) are different. One way to assess the efficiency of cyclic electron flow is to follow the changes in the ETR(I)/ETR(II) ratio. Calculation of ETR(I) and ETR(II) requires the directly measured quantum yield of PSI (Y(I)) and PSII (Y(II)), light intensity and estimated constant factors (see Mat. Meth.). From this definition of ETR it follows that instead of the calculated ETR(I)/ETR(II) ratio the directly measured Y(I)/Y(II) can be used. As shown in Fig. 6, the Y(I)/Y(II) ratio does not show significant differences between the different wheat cultivars either in the control or in drought stress conditions in the biomass accumulation period at various grow light regimes (94–849 µmol photons m^−2^ s^−1^). Only in the well-watered Plainsman V. was the Y(I)/Y(II) ratio somewhat lower under medium and high light regimes (Figs. 6B and 6C). However, the Y(I)/Y(II) ratio increased in drought-stressed Cappelle Desprez cv. during grain filling period at medium (363 µmol photons m^−2^ s^−1^) and high (849 µmol photons m^−2^ s^−1^) light regimes, respectively (Figs. 6B and 6C).

**Figure 7:**
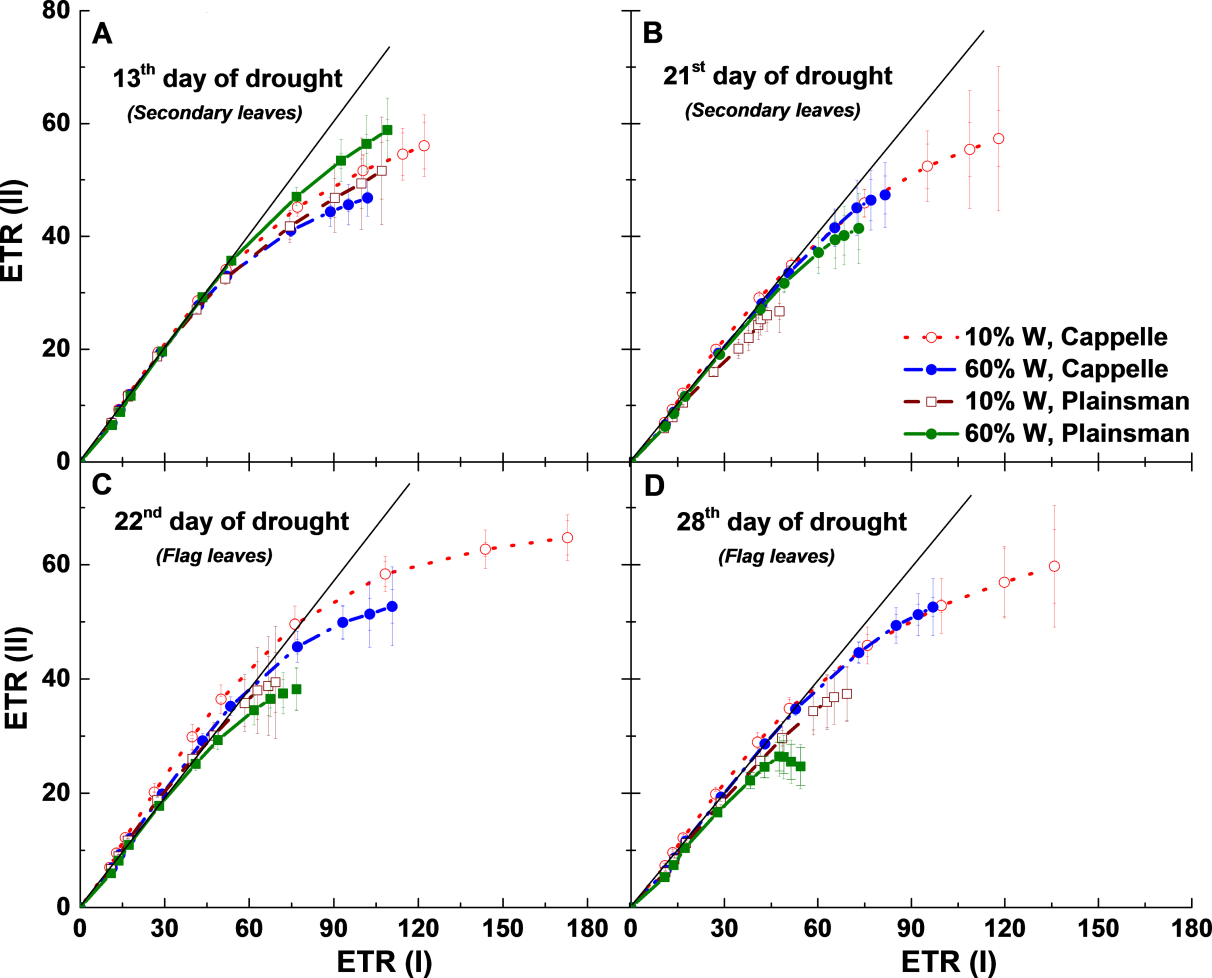
Relationship between PSII and PSI electron transport rates. ETR(II) and ETR(I) were measured as a function of light intensity, and the ETR(II) values were plotted as a function of the corresponding ETR(I) values. Measurements were done in leaves of well-watered (60% W—filled symbols) and drought-stressed (10% W—open symbols) Cappelle Desprez (circles) and Plainsman V (squares) cv. wheat plants, respectively. Data represent the means ± SE of five plants per treatment.

The relationship between ETR (I) and ETR (II) can also be analyzed by looking at the linearity of ETR (II) as a function of ETR (I). When only linear electron flow dominates there is a linear relationship between ETR (II) and ETR (I). However, after the onset of cyclic flow ETR (I) increases faster than ETR (II) and the linear relationship breaks down at high light (Johnson, 2011). During the early days of drought (13th day) cyclic flow sets in under similar conditions, ETR (I) ≈ 60 µmol/m^2^ s, in all treatments (Fig. 7A), but as the drought progresses they show divergent responses. For 21 days of drought there is a tendency for the maximum ETR(I) to increase while the maximum ETR(II) does not change much in the Cappelle cv. (Fig. 7B) implying that linear electron transport is not much affected by the drought treatment while cyclic electron flow around PSI is increasing in the secondary leaves. A Similar tendency is seen in the flag leaves after 22 and 28 days of drought stress (Figs. 7C and 7D). In contrast, drought stressed Plainsman showed a tendency of smaller cyclic flow in the later phases of drought stress (Figs. 7C and 7D). Thus we could observe that as the drought progresses cyclic electron flow is losing in Plainsman, while it is enhancing in Cappelle.

**Figure 8:**
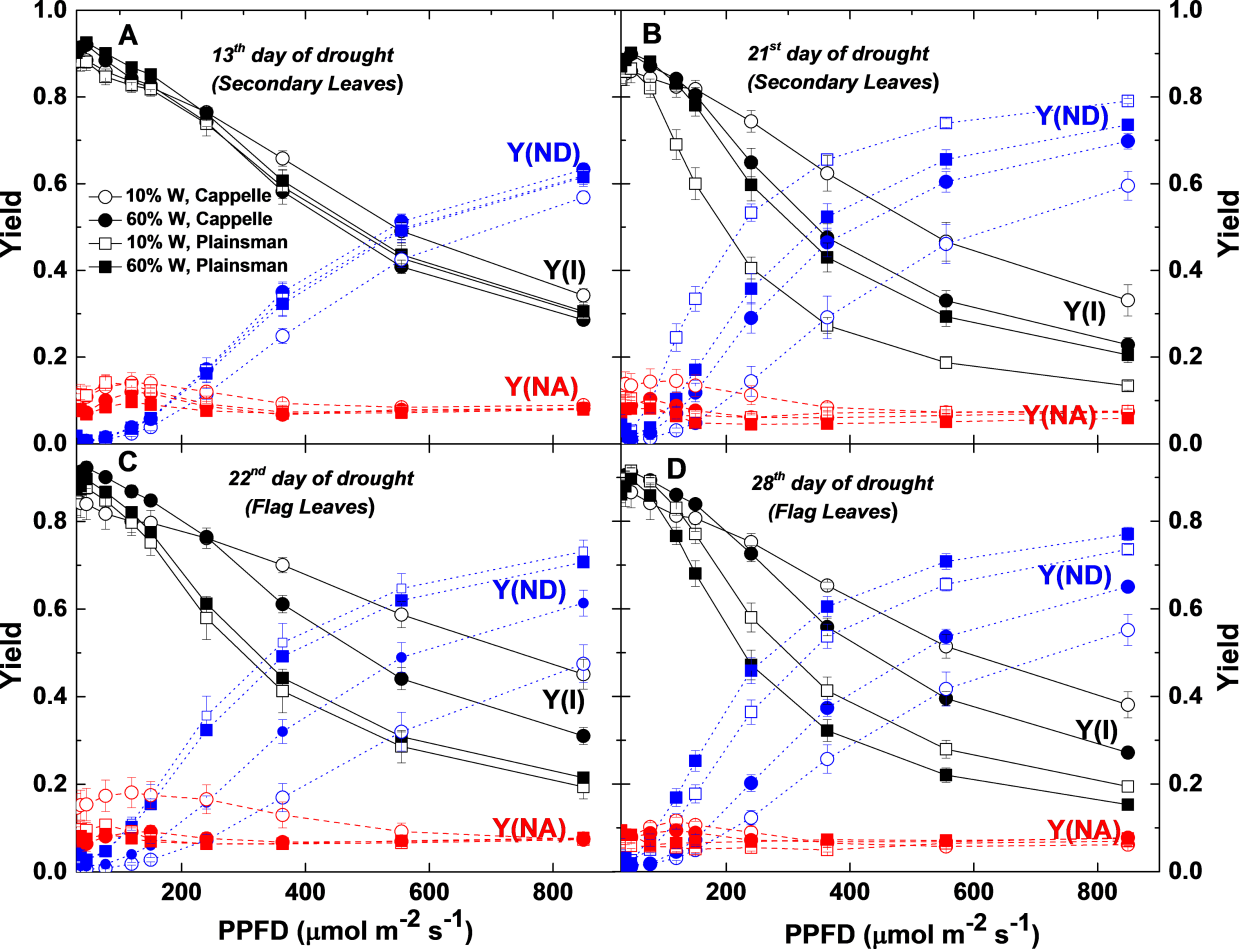
Light response of quantum yield parameters of PSI photochemistry. P700 kinetics were recorded in Cappelle Desprez (circles) and Plainsman V (squares) cv. plants in well-watered (60% W—closed symbols) and drought-stressed (10% W—open symbols) conditions. Y(I), the effective quantum yield of PSI; Y(ND), the quantum yield of non-photochemical energy dissipation due to the donor-side limitation; Y(NA), the quantum yield of non-photochemical energy dissipation due to the acceptor-side limitation are plotted as a function of light intensity. Data represent the means ± SE of five plants per treatment.

### Light responses of the PSI electron transport parameters

Characterization of electron transport through Photosystem I is important for the identification of the sites where limitation of electron flow occurs under conditions of drought stress. Light responses of PSI parameters obtained from P700 signals were further analyzed (Fig. 8). The fraction of overall P700 that is kept in the oxidized state, Y(ND), was significantly increased with the increase of PPFD in secondary and flag leaves of drought stressed Plainsman V from the end of the second week of water withdrawal with respect to Cappelle cv in both 60% and 10% field capacity (Figs. 8B and 8C). Higher values of Y(ND) indicates that a major fraction of overall P700 is in the oxidized state during illumination due to limitation of electron flow from PSII towards PSI under severe drought condition. Y(NA) represents the fraction of overall P700 that cannot be oxidized by a saturation pulse in a given state due to a lack of oxidized PSI acceptors (Singh et al., 2014). A substantial increase of Y(NA) was observed in the flag leaves of drought stressed Cappelle Desprez cv. (Fig. 8C), which correlates with the lower CO_2_ uptake rate of these leaves, that creates a limitation of reduced acceptors at the acceptor side of PSI at lower light intensities (Zivcak et al., 2013). Y(NA) of Cappelle Desprez cv. was greater than Y(ND) at PPFDs <250 µmole photons m^−2^ s^−1^, while, above this level, Y(NA) decreased and Y(ND) of drought-stressed Plainsman V increased. Higher effective quantum yield of PSI, Y(I) was observed for drought-stressed Cappelle Desprez cv. in both biomass accumulation as well as grain filling period in comparison to plants of all other treatment conditions (Figs. 8A–8D).

## Discussion

In the present work we have analyzed the biomass and grain yield accumulation under conditions of drought stress in two model wheat cultivars, Cappelle Desprez and Plainsman V, which are classified in the literature as drought sensitive and tolerant varieties, respectively (Guóth et al., 2009) (http://genbank.vurv.cz/wheat/pedigree) in parallel with the photosynthetic responses of their primary (flag) and secondary leaves. The results obtained from phenotyping for biomass accumulation and grain yield appear to be surprising in the first sight since our findings represent an interesting situation in which green biomass accumulation and grain production respond differentially to water scarcity in different wheat cultivars. Although the “drought sensitive” Cappelle Desprez keeps higher biomass yield than the “drought tolerant” Plainsman V, the latter variety is able to maintain higher absolute grain yield and grain yield stability than the former one under conditions of water limitation. Therefore, one of the important messages of our work is that phenotyping based only on shoot/leaf area (green biomass) can be largely misleading for drawing predictions about grain yield potential under drought, and possibly under other stress conditions.

Similarly to biomass accumulation the water status of the plants also shows differential response to water scarcity. Although both the Cappelle Desprez and Plainsman V cultivars showed similar and rapid initial loss of water from the soil in pots and the water potential in penultimate leaves was significantly decreased in both genotypes during drought stress the extent of reduction of soil water was predominant in the drought sensitive Cappelle Desprez cv. (Guóth et al., 2009). It has also been reported that the relative water content of the flag leaves in the Cappelle Desprez cultivar was decreased significantly during drought stress while in Plainsman V it was not much affected (Fábián et al., 2011; Jäger et al., 2014).

The ultimate source of biomass accumulation is CO_2_ fixed by the photosynthetic apparatus in the form of organic substances. Grain production, which is a specific form of biomass accumulation is driven mostly by the photosynthetic activity of the primary (flag) leaves in wheat plants. The higher CO_2_ uptake rate in the flag leaves of the drought stressed Plainsman V as compared to that of the Cappelle Desprez cv. correlates well with the higher grain yield of the Plainsman V cv. Interestingly, our data also show that the extent of drought induced decrease in CO_2_ accumulation rate was less pronounced for the Cappelle Desprez than for the Plainsman V in the secondary leaves (Fig. 2A), which agrees with the larger green biomass accumulation in the Cappelle Desprez variety. These data together show a differential role of the primary and secondary leaves in biomass accumulation in the form of grains and shoots/leaves, respectively.

Regulation of stomatal function is an important mechanism in dealing with the adverse consequences of drought stress. The typical response of plants to water limitation is stomatal closure through which the amount of water loss via evaporation can be decreased. On the other hand, drought induced closing of stomata limits also CO_2_ uptake; therefore, it decreases the efficiency of net photosynthesis. Since the extent of decreasing the transpiration rate is usually higher than the decrease of net photosynthesis the water-use efficiency (WUE) increases, and the plants conserve water and increase their chances for survival. This typical response can be observed both in the secondary and flag leaves of the Cappelle Desprez cv. (Fig. 2D). The higher WUE values of Cappelle Desprez correlate with its efficiency for delaying senescence (Erdei et al., 2005; Hafsi, Akhter & Monneveux, 2007).

As the drought progresses, the leaf temperature of Cappelle Desprez cv. becomes similar to ambient temperature reflecting stomatal closure. On the contrary, Plainsman V shows prolonged transpiration through flag leaves and spikes during severe drought, which is evident from the cooler than ambient leaf temperatures (Figs. 3C and 3D). This physiological response might be supporting drought tolerant Plainsman V. to regulate the sink region for higher grain yield stability under severe drought.

It is a highly important question: which parameters of photosynthetic electron transport could give information on the tolerance or resistance of the studied varieties to drought stress? According to our data, the *F*_*V*_/*F*_*M*_ ratio did not give information about the extent of resistance (Fig. 5) in agreement with previous literature findings (Lu & Zhang, 1999). However, we could find other deduced variable Chl fluorescence parameters, which are useful for the indication of drought tolerance. Especially the performance index (PI) (Zivcak et al., 2008), Area, RC/ABS and the drought factor index (DFI) parameters developed by Strasser and coworkers (Oukarroum et al., 2007) have proven to be useful indicators of the ability of wheat plants to tolerate the consequences of drought conditions.

The variable Chl fluorescence and P700 kinetics also give valuable information about the effect of drought stress on structural and functional changes of the photosynthetic apparatus. We could observe faster J-I rise in Chl fluorescence transients and faster P700 re-reduction kinetics, which supports the idea that the PSI content is reduced in drought stressed Plainsman V plants in agreement with previous literature data (Joliot & Joliot, 2002; Yan et al., 2013).

Kinetically, the I-P phase has been shown to correlate with PSI activity and changes in the I-P amplitude can be used as semi quantitative indicators for (relative) changes in the PSI content of the leaf (Ceppi et al., 2012). In drought-stressed Cappelle Desprez cv. we could find a consistent high I-P rise phase and low P700^+^ accumulation from the 3rd week of drought stress in secondary leaves to the end of 4th week in flag leaves (Figs. 4B–4D). This effect could be due to enhanced cyclic electron flow around PSI, which is an important defense mechanism against drought and other abiotic stress factors (Joliot & Joliot, 2002; Kono, Noguchi & Terashima, 2014), as a result of a transient block of electron transfer at the electron acceptor side of PSI under a high [NADPH]/ [NADP^+^] ratio (Hamdani et al., 2015).

During light-saturated photosynthesis the excess ETR(I) drives cyclic electron flow (Laisk, Oja & Eichelmann, 2008), which is enhanced under stress conditions, such as drought. In our experiment, we could find that as the severity of drought progressed to the third week (21st–28th day of drought) enhancement of cyclic electron flow through ETR(I) in Cappelle Desprez and losing of ETR(I) in Plainsman V occurred during both biomass accumulation and grain filling period, (Figs. 7B–7D).

### Concluding remarks

Non-invasive photosynthetic measurements provide highly useful tools for making reliable predictions of physiological traits of wheat and other plants. Our findings demonstrate that the agronomically highly important traits of biomass and grain yield are not necessarily correlated in wheat and possibly in other cereal crops. Therefore, phenotyping of biomass responses alone is not sufficient for predictions of grain yield changes. As a consequence, phenotyping protocols should include grain yield assessment when the aim is the optimization of grain yield and grain yield stability under stress conditions.

Our results support the importance of cyclic electron flow in drought stress tolerance. We can also conclude that changes in physiological parameters show different responses to drought stress depending on the developmental stage of leaves in the case of the studied two cultivars. Flag leaves, which serve as grain supporting leaves show similar response in their CO_2_ fixation, drought factor index, and electron transport parameters as the grain yield, whereas the secondary leaves, which support overall green biomass growth show similar responses as biomass accumulation. These findings are warranted by the presented results for the Cappele Deprez and Plainsman V cultivars studied here. However, the general validity of the differential response of physiological parameters in leaves of different developmental stages has to be investigated by using several wheat cultivars in future studies.

## Supplemental Information

10.7717/peerj.1708/supp-1 Supplemental Information 1 Biomass and grain yield For biomass determination digital images of seedlings were taken by using a Nikon D80 camera equipped with an AF-S DX Zoom-NIKKOR 18–135 mm objective (f/3.5-5.6G ED-IF Lens) and close-up rings. Digital images of plant replicates under various drought stress treatments were analyzed for green biomass area using ImageJ software. We used colour thresholding to select just the plant green area and exclude the stand, pot, shadows and yellowish leaves (  http://rsbweb.nih.gov/ij/). Data presented are mean (*n* = 5) ± se of five plants/treatment. Grain yield was determined by collecting seeds and measuring their weight at the and of the growing period.

10.7717/peerj.1708/supp-2 Supplemental Information 2 Chlorophyll fluorescence and P700^+^ kinetics Variable chlorophyll fluorescence transient from PSII and the amount of oxidized PSI primary Chl electron donor (P700^+^) was simultaneously measured using a DUAL PAM-100 system (WALZ, Effeltrich, Germany). *F*(*t*) and *P*(*t*) are the mean values of variable Chl fluorescence and P700^+^ at the indicated time points, calculated from 5 different measurements. The maximum level of P700^+^ is called *P*_*m*_ in analogy with *F*_*m*_. It was determined by application of a saturation pulse (300 ms, 10,000 µmol photons m^−2^ s^−1^; 635 nm).

10.7717/peerj.1708/supp-3 Supplemental Information 3 Gas exchange parameters Gas exchange parameters were measured by using a Licor 6400 gas analyzer (Licor, USA). The gas cuvette conditions were set to 400 ppm CO_2_, ambient temperature and growth light intensity of photosynthetic active radiation (of 400 µmol m^−2^ s^−1^). The listed deduced parameters were calculated by the software of the instrument. Data presented are mean (*n* = 5) of five plants/treatment.

10.7717/peerj.1708/supp-4 Supplemental Information 4 Photosystem I and Photosystem II electron transport rate ratios Electron transport rates through PSI (ETR(I)) and PSII (ETR(II)) were measured in leaves of well watered (60% W-) and drought stressed (10% W) Cappelle Desprez (circles) and Plainsman V. (squares) cv. wheat seedlings at different light intensities of 94, 363 and 849 µmol photons m^−2^s^−1^. The values, which are shown as a function of the duration of drought treatment, represent the mean ±SE of five plants per treatment.

10.7717/peerj.1708/supp-5 Figure S1 Biomass and morphological parameters Biomass and morphological parameters of well watered (60%) and drought stressed (10%) Cappelle Deprez and Plainsmann V wheat plants. The are mean values of three replications with the indicated standard deviation and significance levels.

10.7717/peerj.1708/supp-6 Data S1 Thermal imaging data Thermal images of Cappelle Desprez and Plainsman V. wheat plants were quantified by using ImageJ software by thresholding evaporative cooled area relative to the temperature of the surrounding air. The presented data were obtained from the average of thermal images taken under conditions of water stress (10% W) and well watered control (60% W) on the 20th, 27th and 34th day of drought stress. The means ±SE were calculated from 5 plants/treatment. Representative thermal images of well watered and drought stressed plants are also shown.

10.7717/peerj.1708/supp-7 Figure S2 Grain yield parameters Grain yield parameters of well watered (60%) and drought stressed (10%) Cappelle Deprez and Plainsmann V plants. The data are mean values from 3 replications with the indicated standard deviation.

10.7717/peerj.1708/supp-8 Supplemental Information 5 Photosystem I quantum yield parameters P700 kinetics were recorded in Cappelle Desprez (circles) and Plainsman V. cv. plants in well watered (60% W- closed symbols) and drought stressed (10% W) conditions. Y(I), the effective quantum yield of PSI; Y(ND), the quantum yield of non-photochemical energy dissipation due to the donor-side limitation; Y(NA), the quantum yield of non-photochemical energy dissipation due to the acceptor-side limitation are plotted as a function of light intensity. Data represent the means ±SE of five plants per treatment.

## Additional Information and Declarations

### Data Availability

The following information was supplied regarding data availability: The raw data set is uploaded as Data S1.
