# Supplementary figures and images for: Contrasting response of biomass and grain yield to severe drought in Cappelle Desprez and Plainsman V wheat cultivars
Source: PeerJ. 2016 Feb 18;4:e1708. doi: 10.7717/peerj.1708 (PMC4815492; doi:10.7717/peerj.1708)

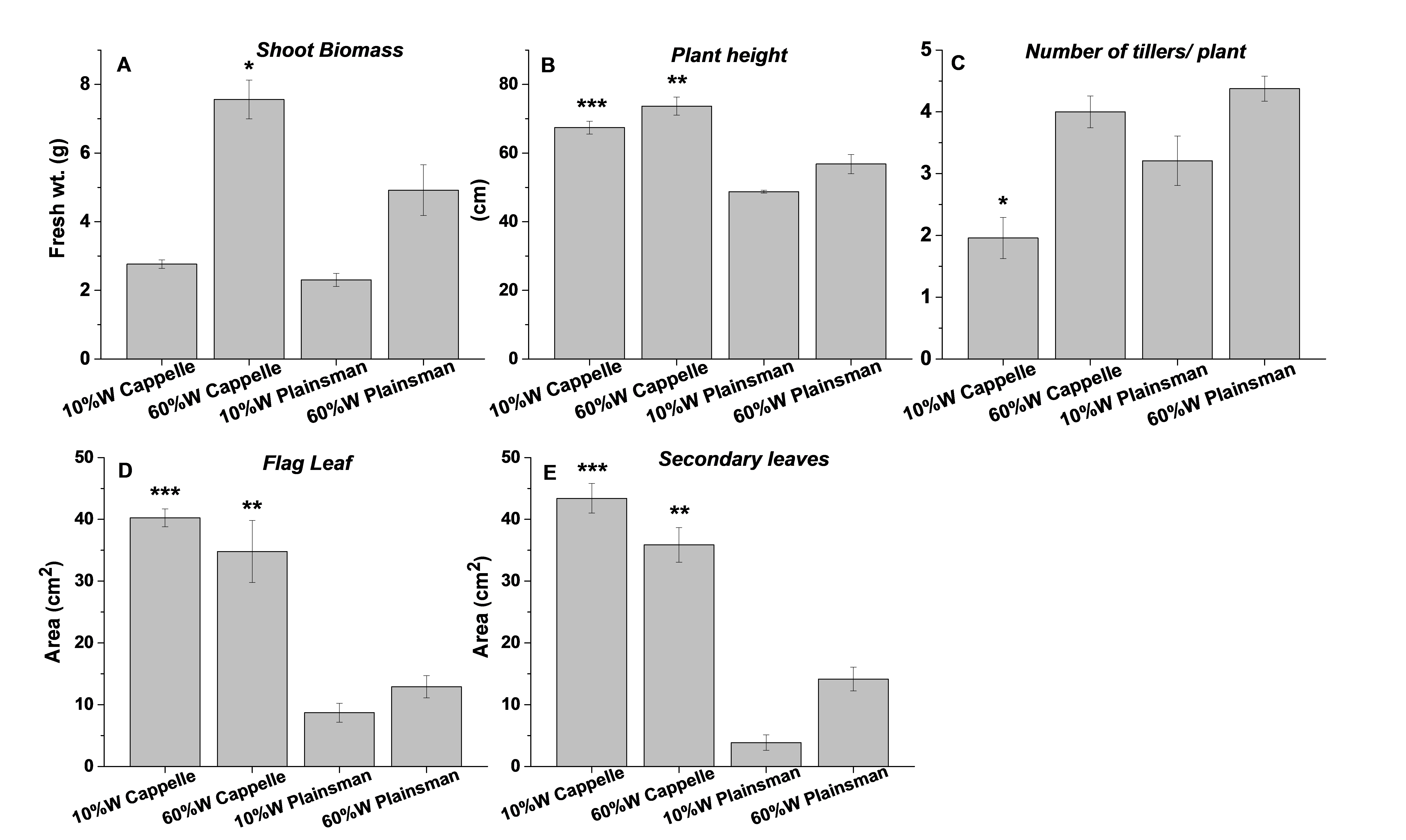

Supplement: Figure S1 — Biomass and morphological parameters of well watered (60%) and drought stressed (10%) Cappelle Deprez and Plainsmann V wheat plants. The are mean values of three replications with the indicated standard deviation and significance levels. [file peerj-04-1708-s005.png]

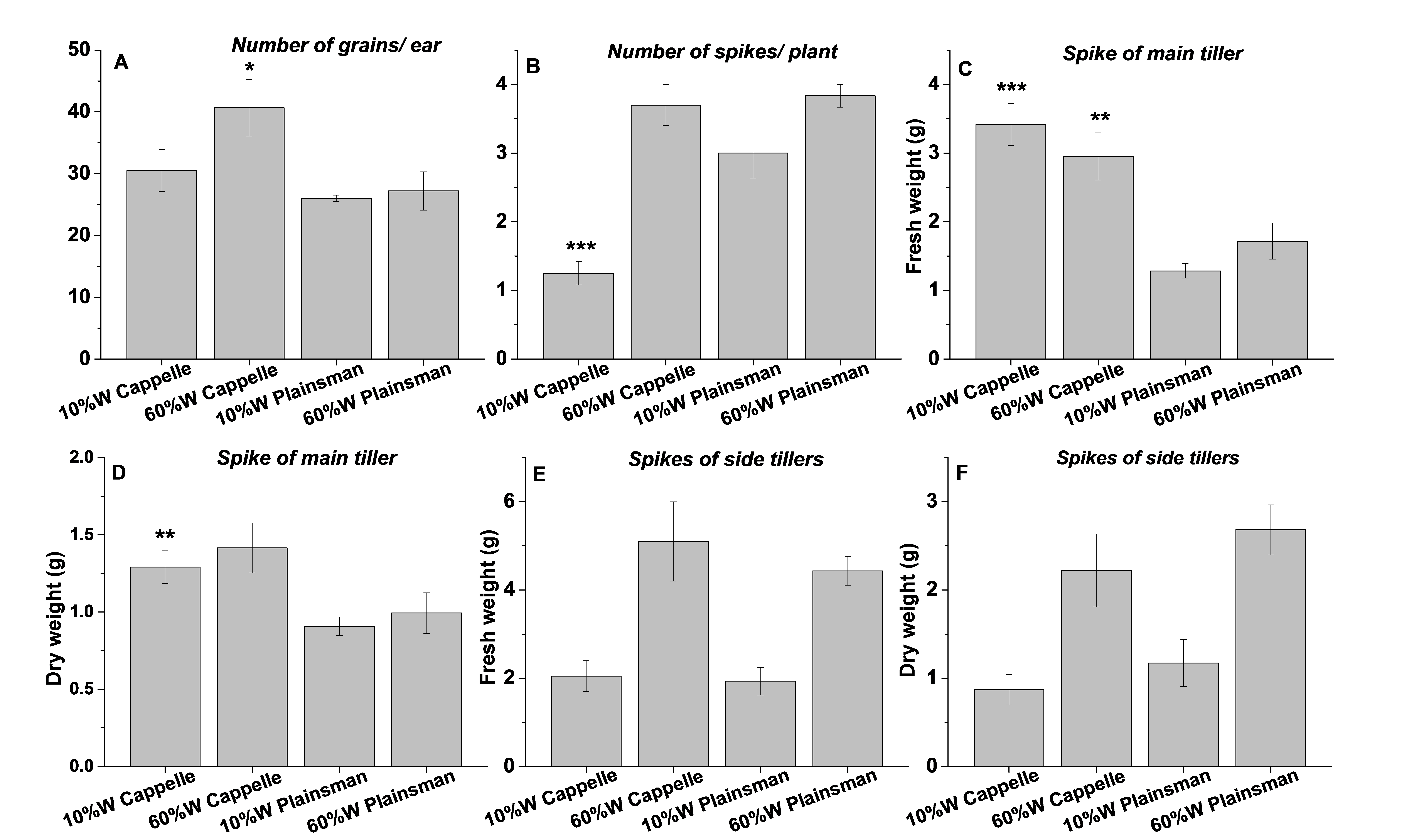

Supplement: Figure S2 — Grain yield parameters of well watered (60%) and drought stressed (10%) Cappelle Deprez and Plainsmann V plants. The data are mean values from 3 replications with the indicated standard deviation. [file peerj-04-1708-s007.png]
